# Machine Learning-Driven Drug Repositioning Identifies Putative IRAK4 Inhibitors Through Structure-Based Computational Evaluation

**DOI:** 10.3390/cimb48090855

**Published:** 2026-08-22

**Authors:** Hyewon Na, Juwon Park, Jiwon Choi

**Affiliations:** College of Pharmacy, Dongduk Women’s University, Seoul 02748, Republic of Korea

**Keywords:** IRAK4, molecular dynamics simulation, MM-PBSA

## Abstract

Interleukin-1 receptor-associated kinase 4 (IRAK4) is one of the IRAK family proteins and plays an important role in the regulation of innate and inflammatory responses. In particular, IRAK4 acts as a key regulator of the Toll-like receptor (TLR) and interleukin-1 receptor (IL-1R) signaling pathways and has attracted attention as a therapeutic target for immune and inflammatory diseases. In this study, an integrated computational approach combining machine learning, molecular docking, and molecular dynamics simulations was applied to identify putative IRAK4 inhibitor candidates. Bioactivity data of IRAK4 were obtained from the ChEMBL and PubChem databases and evaluated for multiple binary classification models. The optimized XGBoost model based on ECFP4 and PubChem fingerprints achieved an ROC-AUC of 0.996 and an average precision (AP) of 0.991 on the independent test set. After that, 20 candidate compounds with high predictive probability score were finally selected through subsequent screening of the DrugBank database. Among them, DB12168 (MK-0557), DB15040 (TP-271), and DB18152 (Zilurgisertib) exhibited favorable binding free energies and stable complex formation with IRAK4 through molecular dynamics simulations and MM-PBSA calculations. Overall, these results demonstrate that approaches incorporating machine learning and structure-based computational analysis can be useful for discovering and prioritizing potential IRAK4 inhibitor candidates.

## 1. Introduction

Inflammation is a fundamental biological response to protect the living body from infection, tissue damage, and other stimuli [1]. These inflammatory responses are primarily mediated by innate immune responses, and the innate immune system recognizes external pathogens and endogenous danger signals through pattern-recognition receptors (PRRs) [1,2]. PRRs recognize pathogen-associated molecular patterns (PAMPs) present in microorganisms and danger-associated molecular patterns (DAMPs) released from damaged tissues. The main PRR families include Toll-like receptors (TLRs), C-type lectin receptors (CLRs), retinoic acid-inducible gene-I-like receptors (RLRs), and nucleotide-binding oligomerization domain-like receptors (NLRs) [3]. In addition, interleukin-1 receptors (IL-1Rs) regulate the inflammatory response by binding to inflammatory cytokines such as IL-1 and initiating signaling within the cell [4]. Among these signaling pathways, most TLRs and IL-1Rs, except for TLR3, transmit signals through myeloid differentiation primary response protein 88 (MyD88) [5]. Both TLRs and IL-1Rs possess a Toll/interleukin-1 receptor (TIR) domain in common, through which MyD88 is recruited to activate the MyD88-dependent signaling pathway, and interleukin-1 receptor-associated kinase 4 (IRAK4) plays an important role in this process [6,7,8]. IRAK4 is a serine/threonine kinase belonging to the IRAK family along with IRAK1, IRAK2, and IRAK3 (IRAK-M), and after receptor activation, it binds to MyD88 via a death domain interaction to form a multiprotein complex called the myddosome. Activated IRAK4 sequentially activates IRAK1 and IRAK2, followed by the recruitment of tumor necrosis factor receptor-associated factor 6 (TRAF6) to activate the nuclear factor-kappa B and mitogen-activated protein kinase 1 signaling pathways [9,10]. Since IRAK4 plays a central role in the early stages of innate immune signaling, its abnormal increase in activity is closely related to the occurrence of various diseases [11,12]. In fact, it has been reported that the hyperactivity of IRAK4 is associated with several types of cancer, as well as autoimmune and inflammatory diseases such as rheumatoid arthritis, systemic lupus erythematosus, psoriasis, and atopic dermatitis [13,14,15]. Therefore, IRAK4 has emerged as an attractive therapeutic target for the treatment of inflammatory diseases, autoimmune diseases, and cancer [16]. Recently, various IRAK4 inhibitors such as Emavusertib (CA-4948), Zimlovisertib (PF-06650833), and KT-474 have been developed and evaluated in the preclinical and clinical trial stages [17,18,19]. These candidates show potential for the treatment of inflammatory diseases and blood cancer, but there are no commercially available IRAK4 inhibitors that have been approved by regulatory agencies to date. Limited clinical efficacy, safety issues, and high heterogeneity among patient groups are known to be major factors that make the development of IRAK4 inhibitors challenging. Therefore, research to discover new IRAK4 inhibitors remains an important task. Alternatively, recent advances in artificial intelligence and machine learning technology are greatly improving the efficiency of the new drug development process. In particular, machine learning-based virtual screening can quickly select promising candidates from large-scale compound libraries, which can significantly reduce the time and cost compared to existing experimental-based exploration processes. In this study, a dataset was constructed by integrating and filtering IRAK4 biological activity data from the ChEMBL and PubChem databases. Based on this dataset, various machine learning classification models were developed, and their performance was evaluated to select the optimal prediction model. The final selected model was applied to the DrugBank database and used for virtual screening and drug repositioning, while molecular docking and molecular dynamics (MD) simulations were carried out on the selected candidates to evaluate the binding stability of the protein–ligand complex. Through this strategy, we tried to assess novel candidate compounds as potential IRAK4 inhibitors for the development of immune-mediated inflammatory disease treatments.

## 2. Materials and Methods

### 2.1. Data Collection and Curation

IRAK4 bioactivity data were collected from the ChEMBL (version 35) and PubChem database, with PubChem data downloaded on 18 April 2025. A total of 6106 candidate compounds for IRAK4 (ChEMBL3778) and 15,321 candidate compounds for IRAK4 (UniProt ID: Q9NWZ3) were obtained from ChEMBL and PubChem, respectively. The collected datasets were preprocessed independently. First, only compounds with IC_50_ values were retained, and records with missing or non-positive activity values (≤0) were removed. The activity units in the PubChem dataset were converted to μM to match those of the ChEMBL dataset. The two datasets were subsequently integrated, and for duplicate compounds with identical canonical SMILES, the lowest IC_50_ value was retained. Finally, 2544 compounds were selected from ChEMBL and 9240 from the PubChem database. To construct a binary classification model, compounds with IC_50_ values ≤ 2 nM were classified as active, whereas those with IC_50_ values ≥ 100 nM were classified as inactive. Compounds with IC_50_ values ranging between 2 and 100 nM were excluded. Finally, a dataset consisting of 2106 active compounds and 4552 inactive compounds was constructed. The data curation process and the number of compounds remaining after each filtering step are summarized in Table 1.

### 2.2. Model Development and Training Strategy

The final dataset (*n* = 6658) was randomly divided into a training set (80%, *n* = 5326) and an independent test set (20%, *n* = 1332) using a fixed random seed (random_state = 42). The test set consisted of 421 active and 911 inactive compounds and was used exclusively for independent model evaluation. To identify the optimal machine learning algorithm for IRAK4 inhibitor prediction, six classification models, including random forest (RF) [20], support vector machine (SVM) [21], extreme gradient boosting (XGB) [22], generalized linear model (GLM) [23], naïve Bayes (NB) [24], and k-nearest neighbors (KNN) [24], were evaluated. For molecular representation, 1304 two-dimensional (2D) descriptors and 881 PubChem fingerprints for 2185 molecular properties were computed using PaDEL-Descriptor software (version 2.21) [25]. Prior to model development, features with zero variance and highly correlated features (r ≥ 0.95) were removed. In addition, the synthetic minority oversampling technique (SMOTE) was applied exclusively to the training set after the training–test split to address class imbalance. Feature scaling was applied only to non-tree-based algorithms, whereas RF and XGB models were trained using the original feature values. After the preprocessing stage, a total of 2410 features were used to build the model. All models were implemented using the scikit-learn (version 1.7.2) [26] and XGBoost libraries (version 3.0.0), and their performance was evaluated using an independent test set. As a result of the evaluation, the XGB model showed the best predictive performance and was selected as the final prediction model. The selected XGB model was constructed by setting the learning rate to 0.05, the number of trees (n_estimators) to 200, and the maximum tree depth (max_depth) to 6, while log-loss was used as the optimization objective. To further assess model robustness against structural overlap, an additional scaffold-based split was performed using Bemis–Murcko scaffolds with a training-to-test ratio of approximately 80:20. Compounds sharing the same scaffold were assigned exclusively to either the training or test set.

### 2.3. Model Evaluation and Performance Metrics

The predictive performance of the model was evaluated using Accuracy, Precision, Recall, F1-score, Area Under the Receiver Operating Characteristic Curve (ROC-AUC), Average Precision (AP), and Cohen’s Kappa (κ) [27]. AP was used to summarize precision–recall performance across different classification thresholds. Accuracy refers to the ratio of correctly classified compounds among the total test data. Precision represents the ratio of actual active compounds among compounds predicted as active compounds, and Recall refers to the ratio accurately predicted by the model among actual active compounds. F1-score was calculated as the harmonic mean of Precision and Recall. Cohen’s Kappa was used to evaluate the degree of agreement between predictive and actual classification results by correcting for coincidence. ROC-AUC was used to evaluate the overall discriminative ability of each model across different classification thresholds. All machine learning analyses and performance evaluations were conducted using the scikit-learn and XGBoost libraries in the Python software environment (version 3.11.8).

### 2.4. Virtual Screening of Drugbank Database

The DrugBank “All Drugs” dataset (version 5.1.13) was downloaded in CSV format and used for machine learning-based virtual screening [28]. The dataset comprised 12,310 entries, including approved, investigational, experimental, nutraceutical, illicit, and withdrawn compounds. To ensure consistency with the training dataset, Extended Connectivity Fingerprints (ECFP4) and PubChem Fingerprints (PubChemFP) were generated for all DrugBank compounds using the same procedures described in Section 2.1. After selecting the XGB model as the final algorithm, additional model optimization was conducted using ECFP4 and PubChemFP. The resulting molecular fingerprints were subsequently used as input features for virtual screening with the final XGB model. Duplicate DrugBank entries were retained because the objective of the virtual screening was to evaluate all available DrugBank records. Therefore, different salt forms, stereoisomers, and formulations were screened as independent entries. The final DrugBank screening dataset contained 12,310 compounds. ECFP4 fingerprints were generated using RDKit [29] with a radius of 2 and a bit size of 2048.

### 2.5. Molecular Docking Procedure

The optimized XGB model was applied to the DrugBank database to predict the probability of IRAK4 inhibition for each compound. Based on the prediction results, the top 20 compounds with the highest predicted probabilities were selected for molecular docking analysis. Three-dimensional (3D) structures of the selected compounds were obtained from the DrugBank database. For compounds lacking 3D coordinates in DrugBank, corresponding 3D structures were retrieved from PubChem. When only two-dimensional (2D) structures were available, 3D conformations were generated and geometry-optimized using Avogadro2 (version 1.102.1). Ligand preparation was performed using AutoDock Tools (v1.5.6), including the addition of hydrogen atoms, assignment of Gasteiger charges, and definition of rotatable bonds. All ligands were subsequently converted into PDBQT format. The crystal structure of IRAK4 complexed with a 4,6-diaminonicotinamide inhibitor (PDB ID: 5W85) was obtained from the RSCB Protein Data Bank. Missing residues in the crystal structure were modeled using MODELLER, and the generated models were evaluated based on the Discrete Optimized Protein Energy (DOPE) score. The 3D structural model with the lowest DOPE score was selected for subsequent docking studies. Protein preparation was performed using AutoDock Tools (v1.5.6) [30]. Water molecules and heteroatoms were removed, polar hydrogen atoms were added, and partial atomic charges were assigned. The prepared protein structure was converted to PDBQT format, and the co-crystallized ligand was extracted and docked as the reference compound. Molecular docking simulations were conducted using AutoDock Vina (v1.1.2) [31]. The docking grid was centered on the ATP binding site (x = 7.70 Å, y = 42.80 Å, z = 9.63 Å) with dimensions of 30 × 30 × 30 Å. Docking was performed for 20 selected compounds and the reference ligand under identical conditions. Compounds exhibiting docking scores comparable to or lower than that of the reference ligand were selected for further molecular dynamics simulations. The binding pose of the four selected compounds were analyzed using the Protein-Ligand Interaction Profiler, to characterize noncovalent interactions and identify major binding residues [32].

### 2.6. Molecular Dynamics Simulations and Trajectory Analysis

Molecular dynamics simulations were performed for five IRAK4–ligand complexes, including the 4,6-diaminonicotinamide reference inhibitor and four selected compounds, using GROMACS (version 2025.3) [33] in conjunction with CHARMM-GUI [34]. The IRAK4 structure prepared for molecular docking (PDB ID: 5W85) was used as the initial protein structure, and protein protonation states and hydrogen-bonding networks were optimized using Protoss (ProteinsPlus) [35]. All ligands were prepared in MOL2 format using Avogadro2 (version 1.102.1) [36], and protein–ligand complex systems were generated using CHARMM-GUI Solution Builder. CHARMM36/CGenFF force field parameters were applied to the protein and ligands. Each complex was solvated in a cubic TIP3P water box with a minimum solute–box boundary distance of 10 Å, resulting in a box dimension of approximately 92 × 92 × 92 Å^3^. Counter ions were added to neutralize the system and to achieve an ionic concentration of 0.15 M NaCl. Each system was first subjected to energy minimization, followed by equilibration under NVT and NPT conditions. During equilibration, the temperature was maintained at 303.15 K using the v-rescale thermostat, and long-range electrostatic interactions were treated using the particle-mesh Ewald (PME) method. Covalent bonds involving hydrogen atoms were constrained using the LINCS algorithm. Production MD simulations were then performed under the NPT ensemble for 50-ns with a 2 fs integration time step. Trajectory coordinates were saved every 25 ps and used for subsequent trajectory analyses. The MD trajectories were evaluated using root mean square deviation (RMSD), root mean square fluctuation (RMSF), radius of gyration (Rg), hydrogen-bond, and molecular mechanics Poisson–Boltzmann surface area (MM-PBSA) analyses. MM-PBSA binding free energy calculations were performed using gmx_MMPBSA (version 1.6.3) [37]. Entropy contributions were not included in the MM-PBSA calculations; therefore, the calculated values were interpreted as relative binding energy estimates rather than absolute binding free energies. For each IRAK4–ligand complex, snapshots were extracted every 25 ps from the entire 50-ns production trajectory (2001 frames). The binding free energy was estimated from the van der Waals, electrostatic, polar solvation, and nonpolar solvation energy components.

## 3. Results and Discussion

### 3.1. Construction of the Irak4 Bioactivity Dataset

**Figure 1:**
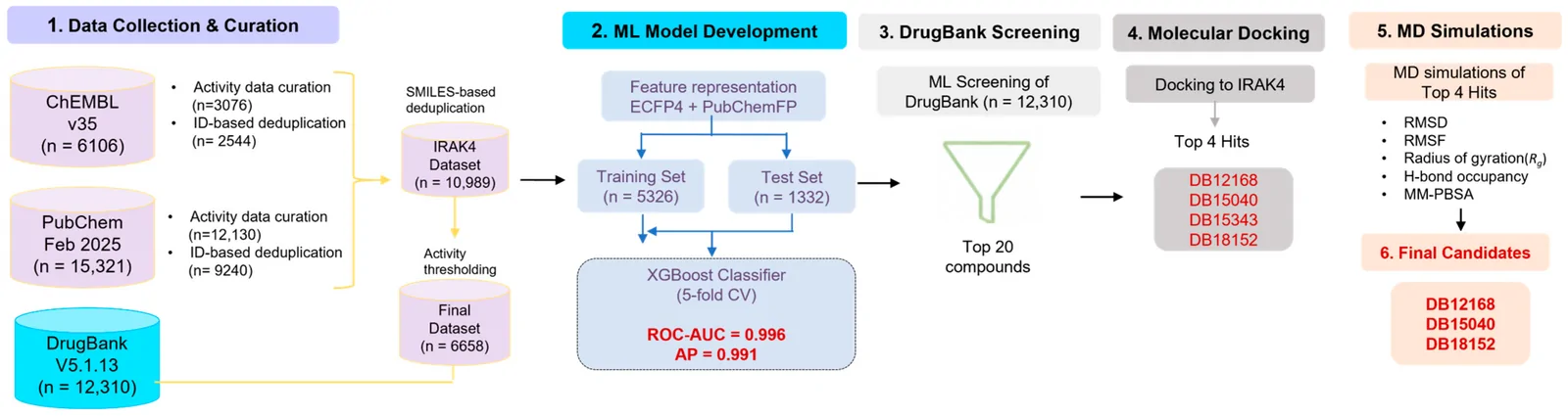
Overall workflow for the identification of potential IRAK4 inhibitor candidates using machine learning-based virtual screening and molecular dynamics simulations. Bioactivity data from ChEMBL and PubChem were used to develop an optimized XGBoost (XGB) model based on ECFP4 and PubChem fingerprints. Following DrugBank screening and structure-based analyses, DB12168, DB15040, and DB18152 were identified as putative IRAK4 inhibitor candidates. Abbreviations: ECFP4, extended-connectivity fingerprint with a radius of 2; XGB, extreme gradient boosting; MD, molecular dynamics.

The bioactivity data for IRAK4 were collected from ChEMBL and PubChem. The dataset consisted of compounds targeting IRAK4, identified by ChEMBL3778 of ChEMBL and UniProt ID Q9NWZ3 of PubChem. The SMILES string was standardized by sequentially performing structural sanitation, aromaticity perception, parent fragments election, charge neutralization, and tautomer canonicalization processes using RDKit (Dec, 2024) [38]. To establish a robust binary classification and reduce label uncertainty of the training set, compounds were categorized using IC_50_ cutoffs. As a result of initial dataset integration, a total of 11,784 unique compounds were obtained. To construct a machine learning model, compounds having an IC_50_ value of 2 nM or less were classified as active, and compounds having an IC_50_ value of >100 nM were classified as inactive. In addition, compounds having an IC_50_ value of >2 nM and <100 nM were excluded to foster a clear distinction between active and inactive compounds during model learning. Finally, a dataset consisting of a total of 6658 compounds including 2106 active and 4552 inactive compounds was constructed. The workflow of this study is summarized in Figure 1.

### 3.2. Machine Learning Model Performance and Selection

**Figure 2:**
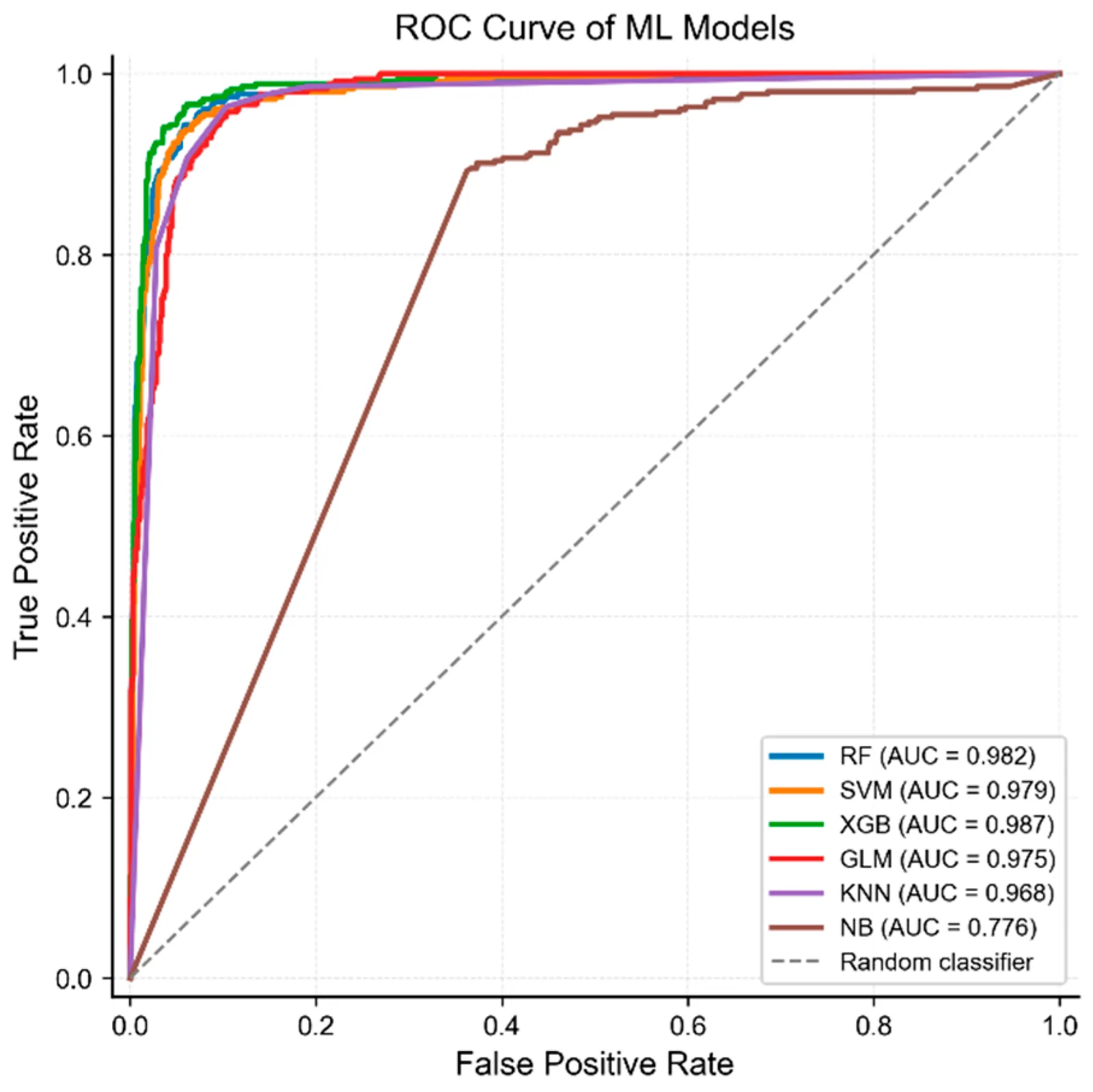
ROC curve analysis of machine learning models for IRAK4 inhibitor prediction. ROC curves were generated for the RF, SVM, XGB, GLM, KNN, and NB models using the independent test set.

To select the optimal machine learning algorithm for IRAK4 inhibitor prediction, the prediction performance of RF, SVM, XGB, GLM, KNN, and NB models was compared using an independent test dataset (Table 2). The final dataset consisted of 2106 active compounds and 4552 inactive compounds, of which 80% were used as training data and the remaining 20% were used as independent test data. The test dataset consisted of 421 active compounds and 911 inactive compounds and was not used in the model training and preprocessing process. All models were evaluated using Accuracy, Precision, Recall, F1-score, ROC-AUC, and Cohen’s Kappa. As a result, the XGB model showed Accuracy (0.960), Precision (0.926), Recall (0.918), F1-score (0.922), ROC-AUC (0.987), and Cohen’s Kappa (0.895) values that showed the best overall performance. In particular, Accuracy and F1-score values were 96.0% and 92.2%, respectively, confirming that active and inactive compounds can be classified with high accuracy. In addition, Cohen’s Kappa value was high at 0.895, confirming that the degree of agreement between the prediction and actual classification results was excellent. Among the remaining models, the RF model recorded an Accuracy of 0.949 and ROC-AUC of 0.982, showing the second-best performance after XGB. The SVM model had a high Recall value of 0.938 and thus had excellent detection ability for active compounds; however, the Precision value was relatively low at 0.834, showing a tendency to increase false positives when predicting active compounds. The GLM model showed relatively stable prediction performance, recording Accuracy of 0.925 and ROC-AUC of 0.975, but the overall performance was somewhat lower than that of the XGB model. The KNN model showed the highest Recall value of 0.963, which allowed effective detection of actual active compounds, but its Precision value showed the lowest level of 0.763, indicating a tendency to misclassify many inactive compounds as active compounds. Therefore, the KNN model showed excellent ability to detect active compounds, but showed limitations in terms of prediction reliability. In contrast, the NB model showed the lowest performance in all evaluation indicators with an Accuracy of 0.646, F1-score of 0.569, and ROC-AUC of 0.776: it was judged to be not suitable for IRAK4 inhibitor prediction. ROC curve analysis results (Figure 2) also supported this trend. Most of the models showed an excellent ROC-AUC value of 0.95 or higher, but the XGB model recorded the highest ROC-AUC value (0.987). This implies that active and inactive compounds can be most effectively distinguished even under various classification threshold conditions. In particular, it was confirmed that the ROC curve was distributed closest to the upper left, thereby securing high sensitivity and specificity at the same time. Overall, the XGB model provided the most balanced results between active compound detection capabilities and predictive reliability. Therefore, the XGB model was selected as the final prediction model for DrugBank database-based virtual screening (Table 3). To further evaluate the robustness and generalizability of the final XGB model, an additional Bemis–Murcko scaffold-based validation was performed. The scaffold-based split retained high predictive performance (ROC-AUC = 0.992 and AP = 0.996), with no scaffold overlap between the training and test sets (Table 4 and Supplementary Table S3).

### 3.3. Feature Optimization of the XGB Model and Virtual Screening of the Drugbank Database

**Figure 3:**
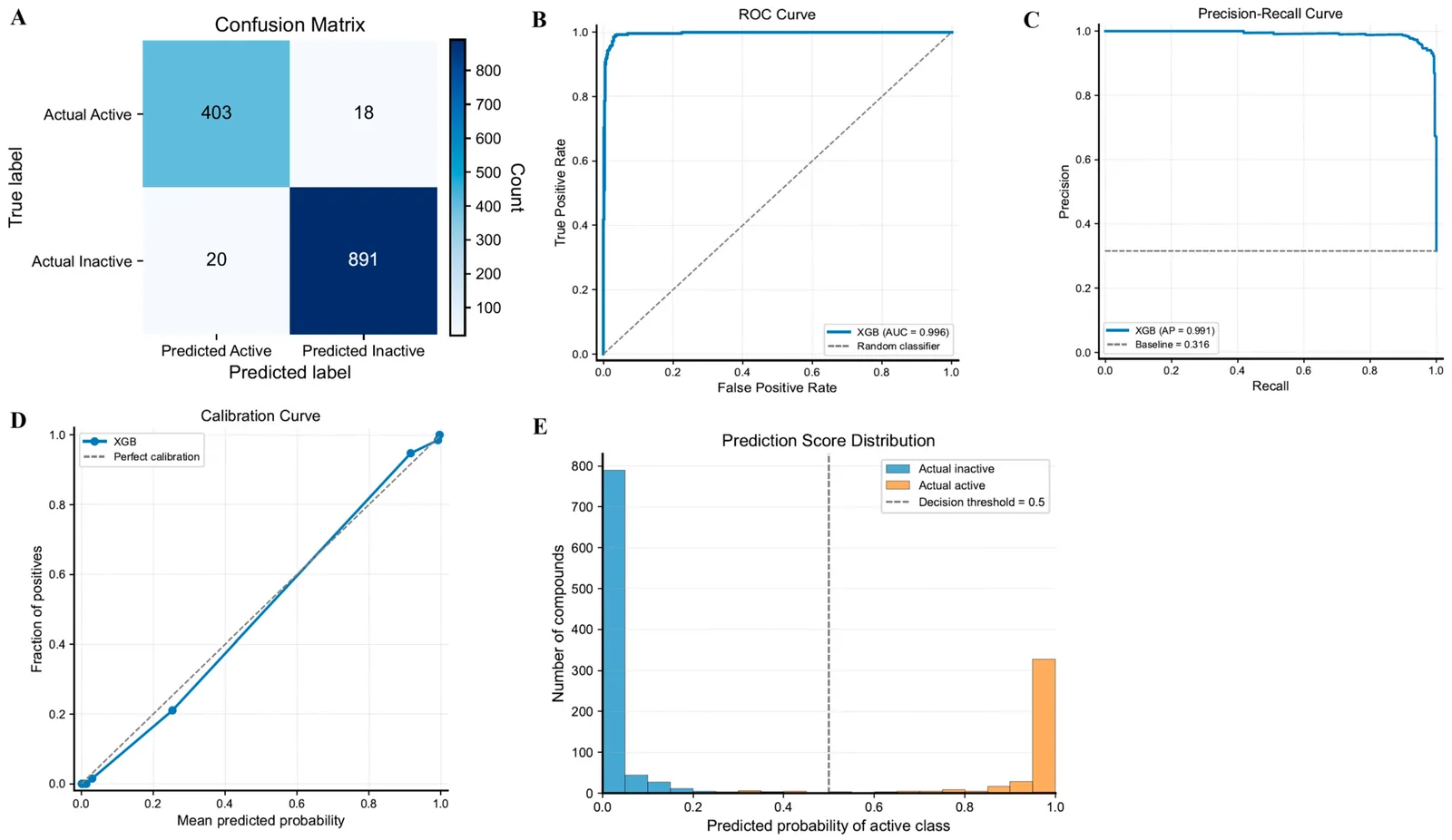
Performance evaluation of the optimized XGB model using ECFP4 and PubChem fingerprints. (**A**) Confusion matrix of the optimized XGB model on the independent test set. (**B**) Receiver operating characteristic (ROC) curve showing an ROC-AUC value of 0.996. (**C**) Precision–recall curve with an average precision (AP) value of 0.991. (**D**) Calibration curve comparing predicted probabilities with observed outcomes. (**E**) Distribution of predicted probabilities for active and inactive compounds at the classification threshold of 0.5.

After the XGB model was selected as the final prediction model, additional feature representation optimization was carried out to improve the prediction performance. In the initial model comparison process, 1304 2D molecular descriptors and 881 PubChemFPs were used but ECFP4 and PubChemFPs were applied in the final model construction stage. As a result, the XGB model with ECFP4 and PubChemFP achieved an accuracy of 0.972, precision of 0.953, recall of 0.957, F1-score of 0.955, ROC-AUC of 0.996, and Cohen’s Kappa of 0.934 on the independent test set (Table 3). In addition, confusion matrix analysis showed that 403 out of 421 active compounds were accurately predicted, and 891 out of 911 inactive compounds were accurately classified (Figure 3A). There were only 18 active and 20 inactive compounds that were misclassified, thus demonstrating the high predictive accuracy of the model. In the ROC curve analysis, the final XGB model also showed a ROC-AUC value of 0.996, confirming excellent discrimination between active and inactive compounds (Figure 3B). The average precision (AP) value was 0.991, maintaining high prediction performance despite the class imbalance in the dataset (Figure 3C). In addition, based on the calibration curve analysis, it was confirmed that the predictive probabilities and observed values were distributed close to the ideal calibration line, so that the model provided reliable probability estimates (Figure 3D). The predictive probability distribution analysis also showed a clear separation between active and inactive compounds based on a classification threshold of 0.5 (Figure 3E). Based on the predicted probabilities and observed outcomes, the XGB model constructed using ECFP4 and PubChemFP was selected as the final virtual screening model. Subsequently, the model was applied to the DrugBank database (version 5.1.13) to search for candidate compounds with a high probability of inhibiting IRAK4. The DrugBank database comprised a total of 12,310 compounds including Approved, Investigational, Experimental, Nutraceutical, Illicit and Withdrawn compounds. We computed ECFP4 and PubChemFP values for all compounds, carried out compound prediction, and selected 20 compounds with a high predicted probability to bind IRAK4, and used them for subsequent molecular docking.

### 3.4. Molecular Docking and Binding Mode Analysis

**Figure 4:**
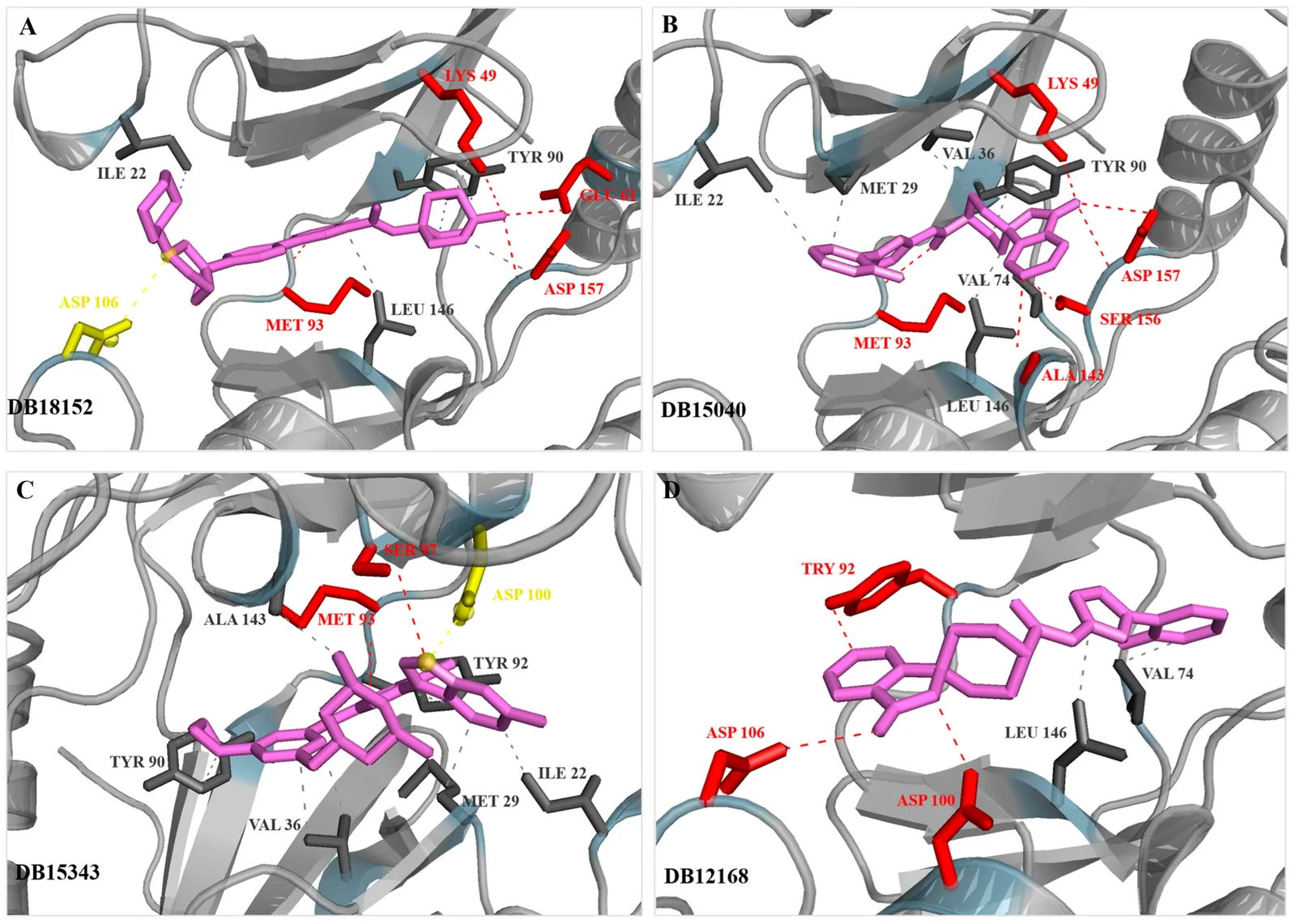
Predicted binding poses of the selected DrugBank compounds within the IRAK4 ATP-binding pocket. (**A**) DB18152, (**B**) DB15040, (**C**) DB15343, and (**D**) DB12168. The IRAK4 protein is shown as a gray cartoon representation, while ligands are displayed as magenta sticks. Hydrogen bonds are represented by red dashed lines, salt bridges by yellow dashed lines, and hydrophobic interactions are indicated by gray dashed lines. The key interacting residues within the IRAK4 binding pocket are labeled.

To evaluate the screening results, molecular docking analysis was performed on the top 20 candidate compounds selected through machine learning-based virtual screening (Supplementary Table S1). As a result, the top four compounds, DB12168, DB15040, DB15343, and DB18152 showed docking scores of −10.5, −10.5, −9.8, and −10.8 kcal/mol, respectively, and displayed better docking scores than the reference compound (−9.4 kcal/mol). Accordingly, these four compounds were selected for subsequent binding mode analysis. Binding mode analysis revealed that all four compounds occupied the ATP-binding pocket of IRAK4 and formed multiple interactions with key active site residues (Figure 4). DB18152 formed a hydrogen bond with Asp157, showed hydrophobic interactions with Met93, Tyr90, and Leu146, and formed a salt bridge with Asp106 (Figure 4A). DB15040 formed a hydrogen bond with Asp157 and Ser156 and showed additional hydrophobic interactions with Ala43, Met93, Val74, and Ile146 (Figure 4B). DB15343 formed a hydrogen bond with Ser97, showed hydrophobic interactions with Tyr90, Tyr92, Met93, and Met129, and formed a salt bridge with Asp100 (Figure 4C). DB12168 formed a hydrogen bond with Asp100 and Asp106, and it was confirmed that the complex was stabilized through hydrophobic interactions with Tyr92, Val74, and Leu146 (Figure 4D). Therefore, these selected compounds have the potential to competitively interact with the ATP binding site and may effectively inhibit IRAK4 activity. As a result, the four selected candidate compounds exhibited favorable binding modes and interaction profiles within the IRAK4 active site and were finally selected for subsequent MD simulations.

### 3.5. Molecular Dynamics Simulation Analysis

**Figure 5:**
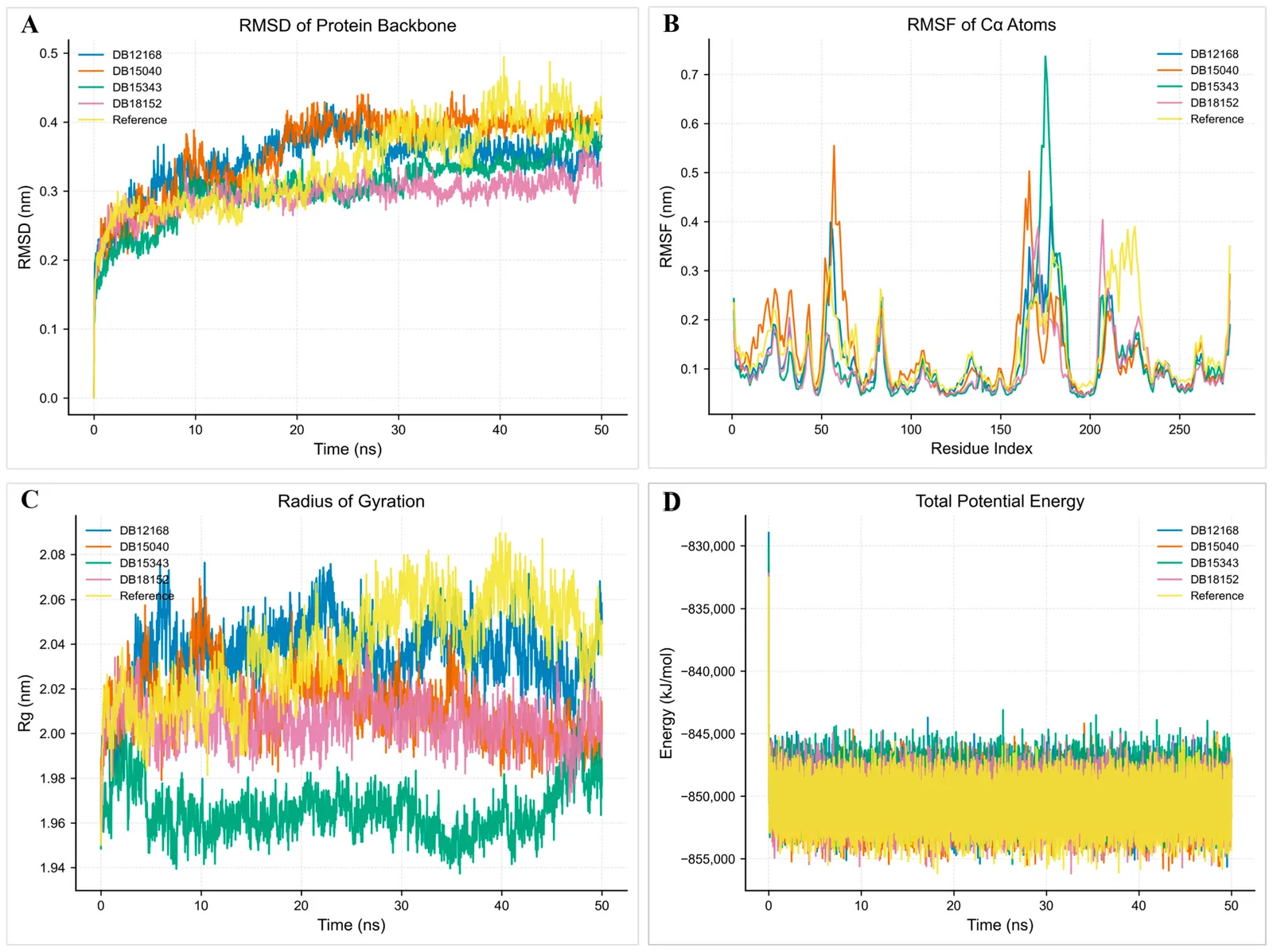
Molecular dynamics simulation analyses of IRAK4 complexes with selected candidate compounds. (**A**) RMSD of the protein backbone, (**B**) RMSF of Cα atoms, (**C**) radius of gyration (*R_g_*), and (**D**) total potential energy profiles during the 50-ns simulation. The reference compound and four selected DrugBank compounds (DB12168, DB15040, DB15343, and DB18152) are shown for comparison. Reference indicates the reference ligand, 4,6-diaminonicotinamide.

**Figure 6:**
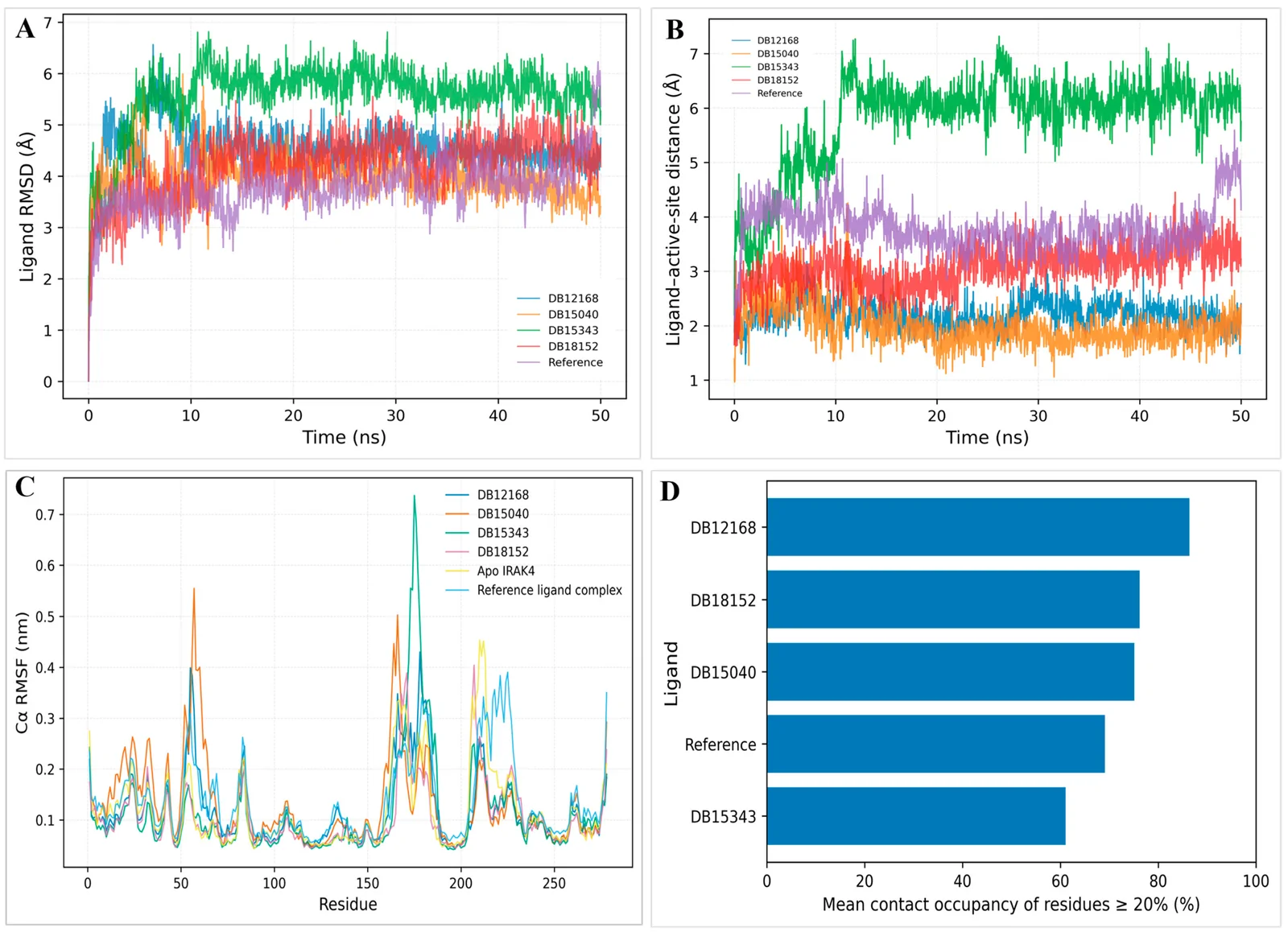
Molecular dynamics analyses of the IRAK4–ligand complexes. (**A**) Ligand RMSD after least-squares fitting to the protein backbone. (**B**) Distance between the ligand and the active-site center during the simulation. (**C**) Backbone Cα RMSF profiles of apo IRAK4 and ligand-bound complexes. The reference ligand complex contains 4,6-diaminonicotinamide. (**D**) Mean occupancy of persistent protein–ligand contacts (residues with contact occupancy ≥ 20%).

The structural stability of the complex was evaluated by performing 50-ns MD simulations on the four selected candidate compounds (DB12168, DB15040, DB15343, and DB18152) and the reference compound (Figure 5). As a result of RMSD analysis, all complexes stabilized rapidly at the beginning of the simulation and then showed a relatively stable behavior in the range of 0.30–0.45 nm (Figure 5A). In particular, the DB18152 and DB12168 complexes represented excellent structural stability by maintaining relatively low RMSD values throughout the simulation. Upon RMSF analysis, most residues showed low variability, while relatively high flexibility was observed only in a specific loop region (Figure 5B). Overall, we found that for the reference compound binding, no rapid modification of the protein structure occurred. Radius of gyration (*R_g_*) analysis confirmed that all complexes remained constant during the simulation, so that the overall structure of the protein remained stable (Figure 5C). In addition, our total potential energy analysis confirmed that the simulation was conducted stably by maintaining a constant energy range for all systems (Figure 5D). Ligand RMSD, ligand–active-site distance, protein Cα RMSF, and protein–ligand contact occupancy consistently indicated that DB12168, DB15040, and DB18152 maintained stable binding throughout the simulations, whereas DB15343 showed greater ligand displacement, increased distance from the binding site, and reduced contact occupancy (Figure 6). Protein residue–ligand contact occupancy and active-site RMSF analyses relative to apo IRAK4 are shown in Supplementary Figure S1. DB12168, DB15040, and DB18152 maintained persistent contacts with multiple active-site residues, whereas DB15343 exhibited fewer sustained residue–ligand contacts. In the H-bond occupancy analysis (Table S2), DB18152 showed high occupancies with Tyr90 (92.1%) and Met93 (69.7%), and DB12168 maintained stable interactions with Met93 (66.5%) and Gly30 (54.1%). DB15040 showed high occupancies with Glu61 (87.8%), and DB15343 showed a relatively high H-bond frequency of interaction with Pro94 (32.2%). Following MM-PBSA analysis, DB15040 (−25.70 kcal/mol), DB12168 (−25.41 kcal/mol), and DB18152 (−24.89 kcal/mol) showed more favorable binding free energy than the reference compound (−18.39 kcal/mol) (Table 5). On the other hand, DB15343 showed a relatively low binding free energy of −10.70 kcal/mol, indicating lower binding stability compared with other candidate compounds. DB12168, DB15040, and DB18152 maintained a stable binding pattern throughout the MD simulation, exhibited favorable binding free energies in the MM-PBSA analysis, and were selected as the final candidate compounds.

Values were obtained from 50-ns molecular dynamics simulations. MM-PBSA ΔGbind values are reported in kcal/mol, where more negative values indicate stronger binding affinity.

## 4. Discussion

In this study, a machine learning-based prediction model was developed using the IRAK4 bioactivity dataset constructed from ChEMBL and PubChem, and novel IRAK4 inhibitor candidate compounds were discovered by applying this machine learning approach to the DrugBank database. The XGB model showed the best predictive performance as a result of comparing various machine learning algorithms, and the final XGB model combining ECFP4 and PubChemFP parameters showed high accuracy and discrimination ability. The XGB model was applied to the virtual screening of compounds. After applying the final XGB model to the DrugBank database, we identified 20 candidate compounds with high predictive probability for IRAK4 binding and carried out molecular docking and MD simulations. As a result, DB12168 (MK-0557), DB15040 (TP-271), DB15343 (Tuspetinib), and DB18152 (Zilurgisertib) were selected as candidate compounds based on their favorable docking scores and binding affinities. Among these candidates, DB12168, DB15040, and DB18152 were considered promising candidate compounds for IRAK4 inhibition due to their stable structural behavior and favorable MM-PBSA binding free energy. However, because entropy contributions were not included, the MM-PBSA results should be interpreted with caution as relative binding energy estimates rather than absolute binding free energies. It should be noted that Zimlovisertib (DB15143), a known IRAK4 inhibitor, was selected as a candidate compound with a high predictive probability value (0.96) from the virtual screening of compounds. This implies that the machine learning model built in this study effectively learned the structural features of IRAK4 inhibitors, showing high reliability. In addition, kinase inhibitors, including Tuspetinib and Zilurgisertib, were also identified as candidates, showing that this model can effectively reflect the chemical properties of the kinase inhibitor family. Meanwhile, MK-0557 and TP-271 have not been reported as IRAK4 inhibitors to date but showed excellent docking scores and stable MD simulation results. This study is meaningful in that novel putative IRAK4 inhibitor candidates were discovered by an integrated computational approach combining machine learning, structure-based virtual screening, and MD simulations based on IRAK4 bioactivity data obtained from public databases. Therefore, it is necessary to validate the IRAK4 inhibitory effect and therapeutic applicability of the candidate compounds identified in this study through biochemical enzyme inhibition experiments and cell-based assays in the future.

## Figures and Tables

**Table 1:** Summary of the data curation process for IRAK4 bioactivity datasets obtained from the ChEMBL and PubChem databases.

Data Curation Step	ChEMBL	PubChem
Initial Dataset	6106	15,321
Activity Data Filtering (IC_50_)	3108	12,162
Missing/Invalid Value Removal	32	32
Deduplication	532	2890
Final Curated Dataset	2544	9240

**Table 2:** Performance comparison of machine learning models for IRAK4 inhibitor prediction using the independent test set.

Model	Accuracy	Precision	Recall	F1-Score	ROC-AUC	Cohen’s Kappa
RF	0.949	0.913	0.887	0.900	0.982	0.866
SVM	0.936	0.834	0.938	0.883	0.979	0.839
XGB	0.960	0.926	0.918	0.922	0.987	0.895
GLM	0.924	0.817	0.910	0.861	0.975	0.810
KNN	0.914	0.763	0.963	0.851	0.968	0.792
NB	0.646	0.413	0.912	0.569	0.776	0.334

Abbreviations: RF, random forest; SVM, support vector machine; XGB, extreme gradient boosting; GLM, generalized linear model; KNN, k-nearest neighbors; NB, naïve Bayes; ROC-AUC, area under the receiver operating characteristic curve.

**Table 3:** Performance comparison of XGB models using different feature representations.

Feature Set	Accuracy	Precision	Recall	F1-Score	ROC-AUC	Cohen’s Kappa
2D Descriptors + PubChemFP	0.960	0.926	0.918	0.922	0.987	0.895
ECFP4 + PubChemFP	0.972	0.953	0.957	0.955	0.996	0.934

Abbreviations: ECFP4, Extended Connectivity fingerprints; PubChemFP, PubChem fingerprints; ROC-AUC, area under the receiver operating characteristic curve.

**Table 4:** Performance comparison of the XGB model using random and scaffold-based data splitting strategies.

Split Strategy	Accuracy	Precision	Recall	F1-Score	ROC-AUC	Cohen’s Kappa	Average Precision (AP)
Random split	0.972	0.953	0.957	0.955	0.996	0.934	0.991
Scaffold-base split	0.974	0.979	0.983	0.981	0.992	0.940	0.996

Abbreviations: AP, average precision; ROC-AUC, area under the receiver operating characteristic curve.

**Table 5:** Summary of molecular dynamics simulation analyses for selected IRAK4 candidate compounds.

Compound	Docking Score (kcal/mol)	Avg. H-Bonds	MM-PBSA ΔG_Bind (kcal/mol)
Reference (4,6-diaminonicotinamide)	−9.4	2.920	−18.39
DB12168	−10.5	1.964	−25.41
DB15040	−10.5	1.574	−25.70
DB15343	−9.8	0.462	−10.70
DB18152	−10.8	2.128	−24.89

## Data Availability

The original contributions presented in this study are included in the article/Supplementary Material. Further inquiries can be directed to the corresponding author(s).

## Supplementary Materials

The following supporting information can be downloaded at: https://www.mdpi.com/article/10.3390/cimb48090855/s1.
