# Supplementary material for: Machine Learning-Driven Drug Repositioning Identifies Putative IRAK4 Inhibitors Through Structure-Based Computational Evaluation
Source: Curr Issues Mol Biol. 2026 Aug 22;48(9):855. doi: 10.3390/cimb48090855 (PMC13605132; doi:10.3390/cimb48090855)
Supplement: Supplementary file 1 [file cimb-48-00855-s001.zip › cimb-4386309-supplementary.pdf]

Machine Learning-Driven Drug Repositioning Identifies Putative IRAK4 Inhibitors through Structure-Based Computational Evaluation

Hyewon Na[1], Juwon Park[1], Jiwon Choi[1,*]

[1]College of Pharmacy, Dongduk Women's University, Seoul 02748, South Korea

*** Correspondence:**
Jiwon Choi
edccjw@gmail.com

Supplementary Table S1. Molecular docking results of the top 20 compounds selected from machine learning-based virtual screening against IRAK4. The top 20 compounds with Predicted probability ≥ 0.5 were subjected to molecular docking analysis. Predicted probability, docking score, and generic name are presented for each compound. Four compounds selected for subsequent binding mode and molecular dynamics simulations (DB12168, DB15343, DB15040, and DB18152) are highlighted in bold.

Rank	DrugBank ID	Predicted Probability	Docking Score (kcal/mol)	Generic Name
1	DB18917	0.993375	-7.6	Edecesertib
2	DB18749	0.964180	-8.7	Soquelitinib
3	DB15143	0.958279	-8.7	Zimlovisertib
4	DB18823	0.938076	-9.4	Zemirciclib
5	DB15401	0.921285	-8.9	Danicopan
**6**	**DB12168**	**0.822366**	**-10.5**	**MK-0557**
**7**	**DB15343**	**0.769910**	**-9.8**	**Tuspetinib**
**8**	**DB15040**	**0.725500**	**-10.5**	**TP-271**
9	DB11951	0.710189	-9.5	Lemborexant
10	DB16228	0.689624	-8.2	Mavelertinib
11	DB12876	0.660844	-8.5	GS-9256
12	DB14867	0.647994	-8.2	Parsaclisib
13	DB01208	0.646849	-8.7	Sparfloxacin
14	DB11713	0.635511	-8	Ruzasvir
15	DB19214	0.616656	-6.9	LB-102
16	DB18768	0.615332	-8.2	Yimitasvir
17	DB19137	0.598639	-8.5	Batoprotafib
18	DB17560	0.598324	-9.8	Rineterkib
19	DB19218	0.585402	-7.7	Camizestrant
**20**	**DB18152**	**0.569974**	**-10.8**	**Zilurgisertib**

Supplementary Table S2. Hydrogen-bond occupancy of ligand–IRAK4 interactions during the 50-ns MD simulations. Only hydrogen bonds with occupancies ≥20% are listed.

Ligand	Residue	Occupancy (%)
Reference	ASP157	80.4
Reference	TYR92	55.1
Reference	MET29	54.5
Reference	SER156	22.3
DB12168	MET93	66.5
DB12168	GLY30	54.1
DB12168	TYR92	47.1
DB12168	SER97	22.9
DB15040	GLU61	87.8
DB15040	SER156	33.3
DB15040	GLY96	29.4
DB18152	TYR90	92.1
DB18152	MET93	69.7
DB15343	PRO94	32.2

Supplementary Table S3. Bemis–Murcko scaffold composition of the training and test sets used for scaffold-based validation.

Ligand	Occupancy (%)
Unique Bemis–Murcko scaffolds in the full dataset	2493
Unique scaffolds in the training set	1920
Unique scaffolds in the test set	573
Shared scaffolds between the training and test sets	0
Scaffold overlap between training and test sets (%)	0

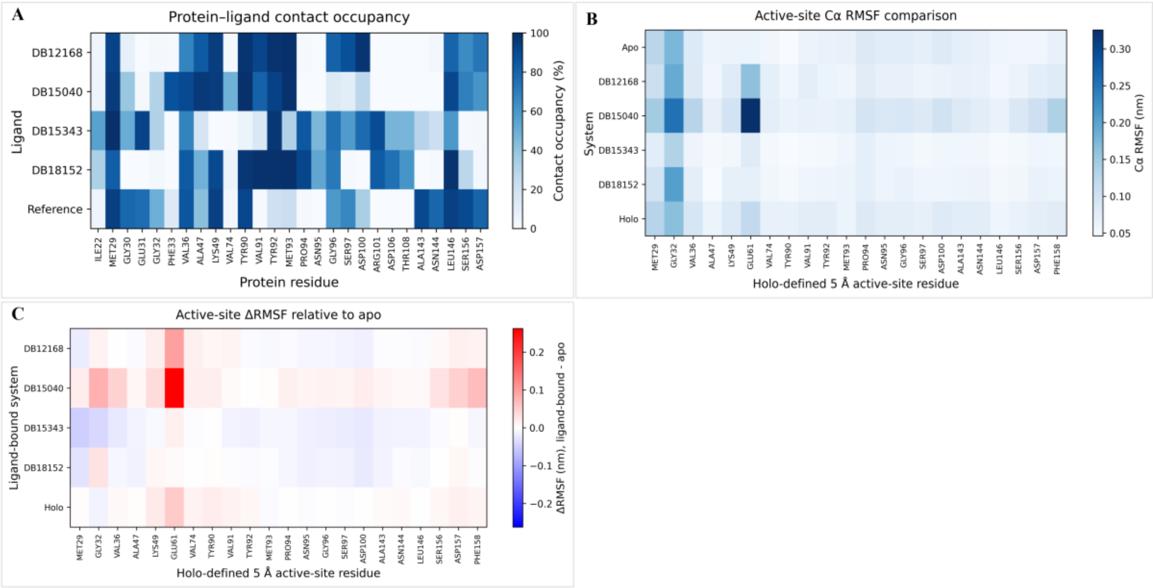

Figure S1. Residue-level analyses of IRAK4–ligand interactions during the 50-ns molecular dynamics simulations. (A) Protein residue–ligand contact occupancy heatmap showing the percentage of simulation time that each residue remained in contact with the ligand. (B) Comparison of active-site Cα RMSF values for apo IRAK4, holo IRAK4, and ligand-bound complexes. Active-site residues were defined as residues located within 5 Å of the co-crystallized ligand in the holo IRAK4 structure. (C) Active-site ΔRMSF relative to apo IRAK4 (ligand-bound minus apo), highlighting ligand-induced changes in local residue flexibility.
